# Multiple relapses of *Plasmodium vivax* malaria acquired from West Africa and association with poor metabolizer CYP2D6 variant: a case report

**DOI:** 10.1186/s12879-019-4357-9

**Published:** 2019-08-09

**Authors:** Xi He, Maohua Pan, Weilin Zeng, Chunyan Zou, Liang Pi, Yucheng Qin, Luyi Zhao, Pien Qin, Yuxin Lu, J. Kevin Baird, Yaming Huang, Liwang Cui, Zhaoqing Yang

**Affiliations:** 1 Department of Pathogen Biology and Immunology, Kunming Medical University, Kunming, Yunnan 650500 People’s Republic of China; 2 Shanglin County People’s Hospital, Shanglin, Guangxi 530500 People’s Republic of China; 3 Guangxi Zhuang Autonomous Region People’s Hospital, Nanning, Guangxi 530021 People’s Republic of China; 4 Guangxi Zhuang Autonomous Region Center for Disease Prevention and Control, Nanning, Guangxi 530021 People’s Republic of China; 5 Eijkman-Oxford Clinical Research Unit, Jalan Diponegoro No. 69, Jakarta, 10430 Indonesia; 6 Centre for Tropical Medicine and Global Health, Nuffield Department of Medicine, University of Oxford, Old Road Campus, Roosevelt Drive, Oxford, OX3 7FZ UK; 7 Department of Internal Medicine, Morsani College of Medicine, University of South Florida, 3720 Spectrum Blvd, Suite 304, Tampa, FL 33612 USA

**Keywords:** *Plasmodium vivax*, West Africa, Relapse pattern, Primaquine failure, CYP2D6

## Abstract

**Background:**

*Plasmodium vivax* transmission in West Africa, dominant for the Duffy-negative blood group, has been increasingly recognized from both local residents as well as international travelers who contracted *P. vivax* malaria there. However, the relapsing pattern and sensitivity to antimalarial treatment of *P. vivax* strains originated from this region are largely unknown. There is evidence that the efficacy of primaquine for radical cure of relapsing malaria depends on host factors such as the hepatic enzyme cytochrome P450 (CYP) 2D6.

**Case presentation:**

A 49-year-old Chinese man was admitted to the Shanglin County Hospital in Guangxi Province, China, on December 19, 2016, 39 days after he returned from Ghana, where he stayed for one and a half years. He was diagnosed by microscopy as having uncomplicated *P. vivax* malaria. Treatment included 3 days of intravenous artesunate (420 mg total), and 3 days of chloroquine (1550 mg total), and 8 days of primaquine (180 mg total). Although parasites and symptoms were cleared rapidly and he was malaria-negative for almost two months, he suffered four relapses with relapse intervals ranging from 58 to 232 days. The last relapse occurred at 491 days from his first vivax attack. For the first three relapses, he was treated similarly with chloroquine and primaquine, sometimes supplemented with additional artemisinin combination therapies (ACTs). For the last relapse, he was treated with intravenous artesunate, 3 days of an ACT, and 7 days of azithromycin, and had remained healthy for 330 days. Molecular studies confirmed *P. vivax* infections for all the episodes. Although this patient was diagnosed to have normal glucose-6-phosphate dehydrogenase (G6PD) activity, his CYP2D6 genotype corresponded to a *2A/*36 allele variant suggesting of an impaired primaquine metabolizer phenotype.

**Conclusions:**

This clinical case suggests that *P. vivax* malaria originating from West Africa may produce multiple relapses extending beyond one year. The failures of primaquine as an anti-relapse therapy may be attributed to the patient’s impaired metabolizer phenotype of the CYP2D6. This highlights the importance of knowing the host G6PD and CYP2D6 activities for effective radical cure of relapsing malaria by primaquine.

## Background

*Plasmodium vivax* is geographically the most widely distributed malaria parasite, and was responsible for about 14.3 million malaria cases in 2017 in the world [1]. Malaria in Southeast Asia and South America is dominated by *P. vivax*; in Africa, *P. vivax* is much less common than the overwhelming number of *P. falciparum* cases. Historically, it has been considered extremely rare or “absent” in Central and West Africa because of the dominance of the Duffy-negative blood group, which was regarded as a receptor required for the invasion of the erythrocytes by *P. vivax* [2]. In recent years, there is growing evidence documenting *P. vivax* infections in local residents of Central and West Africa as well as international travelers returning from the regions to malaria-free countries [3, 4]. In China, for example, of the 909 imported *P. vivax* cases in 2012, 122 originated in 21 Central and West African countries [5]. These records provide compelling evidence of widespread *P. vivax* transmission in Africa. Notably, the identification of *P. vivax* strains able to infect Duffy-negative individuals [6–8] demonstrates that Duffy negativity is not a definitive barrier to vivax transmission. In addition, the presence of very few Duffy-positive hosts in these populations may be enough to sustain low-level transmission of *P. vivax* malaria in these regions. The considerable number of vivax cases routinely diagnosed among Duffy-positive international travelers acquired in West and Central Africa implies hidden local transmission of *P. vivax* malaria in these regions despite predominance of Duffy negativity.

Historically, *P. vivax* malaria has been called a “benign tertian malaria,” but this is a misnomer, given that recent studies have demonstrated severe and fatal pathologies associated with *P. vivax* malaria [9, 10]. Relapses caused by the liver hypnozoites of this parasite can occur months or even several years after the primary attack, thereby contributing to additional morbidity and potentially onward transmission that impedes malaria elimination. In Papua New Guinea, it was estimated that 80% of acute incident vivax infections was derived from relapses rather than new mosquito-borne infections [11].

In most *P. vivax*-endemic areas, chloroquine/primaquine (CQ/PQ), offering blood schizontocidal and hypnozoitocidal therapy, has been used for more than 60 years and remains the frontline radical cure for uncomplicated vivax cases in most regions (excepting Southeast Asia where resistance to chloroquine commonly occurs) [12]. The standard treatment regimen includes three days of CQ (total 25 mg/kg) plus fourteen daily dosages of PQ (0.25 mg/kg). However, PQ imposes a serious problem of toxicity in patients with glucose-6-phosphate dehydrogenase (G6PD) deficiency, an X-linked enzymopathy that can be highly prevalent in endemic areas. As a result, without testing G6PD status of the patients, health providers often do not offer PQ for fear of potentially severe hemolysis. When PQ is prescribed without direct supervision, adherence to the 14-day regimen is poor [13]. To improve adherence, China recommends a shorter 8-day course of PQ regimen (0.375 mg/kg/day; total dose of 3.0 mg/kg) for relapsing malaria (http://www.chinacdc.cn/tzgg/201109/t20110906_52137.htm). Although the 8-day PQ regimen has not been rigorously tested in clinical trials, earlier studies indicate that the temperate *P. vivax* strains from Korea are completely susceptible to a total dose of 3.5 mg/kg [14].

Another problem that affects the effectiveness of PQ for radical cure of vivax malaria involves the host hepatic enzyme cytochrome P450 (CYP) 2D6 [15], which mediates activation of PQ to its active metabolite(s) [16, 17]. CYP2D6 is involved in the metabolism of > 20% prescribed medicine. It is noteworthy that CYP2D6 is the member of the CYP450 family having the greatest genetic polymorphism [18, 19]. The frequencies of alleles with reduced function are as high as 50% in some Asian populations [20], although contributions of different CYP2D6 alleles to PQ failures still need to be firmly established [21, 22]. In clinical trials to assess the effectiveness of PQ for preventing relapses, even under directly observed high-dose PQ therapy, as many as 13.9% *P. vivax* patients experienced relapses [23, 24]. Using genotyping and dextromethorphan metabolism phenotyping of CYP2D6 polymorphism in those subjects, Baird et al. documented almost all of those treatment failures to be associated with impaired CYP2D6 function [25].

Here we describe multiple relapses of a case of vivax malaria acquired in West Africa that also appears to be associated with impaired CYP2D6 metabolism of PQ.

## Case presentation

### Case

A 49-year-old man who had experienced fever and chills for half a day was admitted to the Shanglin County Hospital in Guangxi Province, China, on December 19, 2016. He showed additional symptoms including headache, body aches and cough (Table 1). Upon inquiry of travel history, he informed the doctor that he had spent one year and three months in Ghana (8/15/2015–11/10/2016) and returned home 39 days ago. He explained that during his stay in Ghana, he had experienced two episodes of malaria (species unknown); his last episode of malaria was about half a year ago and both times he was self-treated with artemisinin drugs. On admission, he weighed of 70.2 kg, his axillary temperature was 38.0 °C, and his heart rate, blood pressure and respiratory rate were 92 beats/min, 91/60 mmHg and 20 breaths/min, respectively. With his recent travel history, venous blood was drawn for general hematology and blood chemistry analyses, and a drop of the blood was used to make a thin smear for malaria diagnosis by microscopy. Microscopic examination of the Giemsa-stained blood smear revealed *P. vivax* parasites. Blood tests showed increases in white blood cells (14.25 × 10^9^/L; reference range 4–10 × 10^9^), neutrophil ratio (85.0%; 43–76%), and C-reactive protein (142.29 mg/L). He was conscious and oriented to time, place, and person. He was not dehydrated, pale, or in respiratory distress. Since he did not have any severe symptoms, he was diagnosed as having uncomplicated vivax malaria. He spent three days in the county hospital and was given three days of oral CQ therapy (total 1550 mg). To help resolve the symptoms faster, he was also given intravenous (IV) injections of artesunate (total dose of 420 mg, initial dose of 120 mg, subsequently divided into 5 times of 60 mg at a 12 h interval). Meanwhile, after confirming that he is G6PD normal, an 8-day course of PQ (22.5 mg/day) was begun. Fever was cleared within one day and parasitemia was cleared within two days. The patient was discharged on day four with instructions for follow-up visits if symptoms reappear. Administration of the remaining five days of PQ was directly observed therapy (DOT) by local Center for Disease Control (CDC) personnel to ensure compliance.

**Table 1:** The timeline of the *P. vivax* attacks for the patient and antimalarial treatment

	1st attack	2nd attack	3rd attack	4th attack	5th attack
Days from previous attack	0	58	113	88	232
Days from the 1st attack	0	58	171	259	491
Axillary temperature (°C) on admission	38.0	38.3	38.4	38.6	38.9
Symptoms^a^	R, H, B, C	R, H, B	R, H, B	R, H, B	R, H, B
Parasitemia (%) on admission	0.36	0.25	0.19	0.18	0.38
Treatment^b^	CQ/PQ + AS (420 mg)	CQ/PQ + AS (660 mg); ACT1	CQ/PQ + AS (660 mg); ACT2	CQ/PQ; ACT2	AS (660 mg) + AZ (3500 mg); ACT2
Fever clearance time (h)	16	21	18.5	NA	23

^a^Symptoms: *R* – rigor, *H* – headache, *B* – body aches, *C* – cough

^b^Drug treatment: CQ/PQ – chloroquine (total 1200 mg base, 600 mg on day 1, 300 mg each on day 2 and 3)/primaquine (total 180 mg, 22.5 mg daily for 8 days), *AS* – artesunate at 12 h interval, *ACT1* – artesunate-amodiaquine, *ACT2* - dihydroartemisinin-piperaquine, *AZ* – azithromycin, *NA* – not available

The second attack of febrile paroxysm occurred 58 days later on February 15, 2017, and he was admitted to the county hospital again with similar symptoms as the first attack and diagnosed with *P. vivax* malaria by microscopy (Table 1). The patient had not left Guanxi Province during the 58 days between these two attacks. He was hospitalized for six days and was treated with the same CQ/PQ combination, together with eleven IV injections of artesunate (total dose of 660 mg at an interval of 12 h). Given that the doctor was not sure whether this re-occurrence was due to chloroquine resistance or potential mixed infection with *P. falciparum*, at discharge he was given additional three days of an artemisinin-based combination therapy (ACT), artesunate-amodiaquine, which has a different aminoquinoline drug. Both ACT and PQ were administered as DOT by local CDC staff.

One hundred and thirteen days later, on June 8, 2017, he suffered a third attack of confirmed *P. vivax* malaria and hospitalized at the county hospital for six days. He received the same therapy as for the second attack including DOT of 8-day PQ. At home, he was further treated with three days of a different ACT, dihydroartemisinin-piperaquine.

Eighty-eight days later, on September 4, 2017, he suffered a fourth attack of confirmed vivax malaria. This time he was not hospitalized, while the same CQ/PQ regimen together with three days of oral therapy of dihydroartemisinin-piperaquine was prescribed. All treatments were taken at home and supervised by local CDC staff.

Despite the fact that, after returning from Ghana, this patient lived the entire time in a malaria-free area, he had the fifth attack of vivax malaria 232 days later, on April 24, 2018, 491 days from the first attack. He was admitted to the county hospital for three days and was given IV injection of artesunate six times at a 12 h interval (120 mg each at the first three injections, and 60 mg each at the three subsequent injections). PQ was not prescribed since it was judged not effective. Instead, he was treated with azithromycin (500 mg/day) for seven days. At discharge, he was also given three additional days of dihydroartemisinin-piperaquine. At the time of this interview, he had remained healthy for 330 days after this last episode of vivax malaria.

### Laboratory studies

Venous blood was collected at the time of diagnosis at the first, second, third, and fifth attacks. Blood samples were used for molecular diagnosis and genotyping at the Kunming Medical University laboratory. For each sample, total DNA was extracted from 0.2 mL of venous blood using the High Pure PCR Template Preparation Kit (Roche, Switzerland) following the manufacturer’s instruction and eluted in 100 μl of water. *Plasmodium* species were identified by nested PCR targeting the 18S rRNA genes using genus-specific and species-specific primers for *P. falciparum*, *P. vivax*, *P. malariae* and *P. ovale* [26]. The PCR results showed that all the samples were positive only for *P. vivax* (data not shown).

**Fig. 1:**
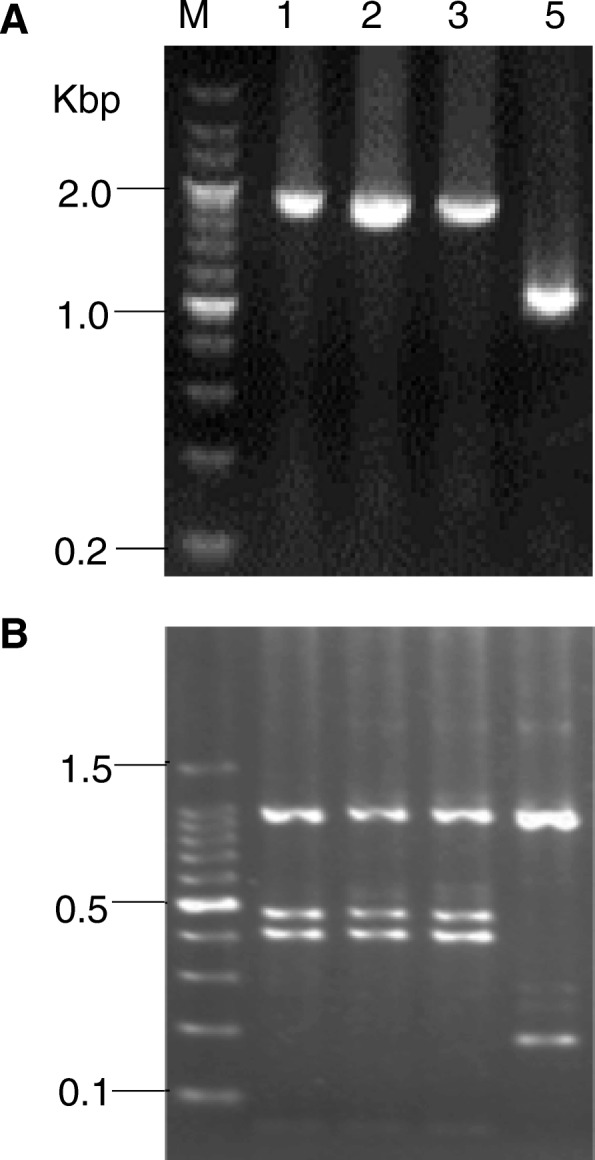
Genotyping of *P. vivax* parasites in primary and subsequent attacks of the same patient. **a** Nested PCR analysis of *PvMSP3α* gene from the primary *P. vivax* infection (lane 1) and subsequent relapses (lanes 2, 3, and 5). **b** Restriction digestion of the nested PCR products shown in panel A by *Hha*I. M, molecular marker in Kbp

To determine whether the relapses were caused by different parasite strains, we genotyped the polymorphic *P. vivax merozoite surface protein (PvMSP) 3α* gene by the nested PCR and restriction fragment length polymorphism (PCR/RFLP) methods described earlier [27]. PCR of *PvMSP3α* alone detected a similar band size for the first three attacks, but the PCR product from the fifth attack was smaller (Fig. 1a). Digestion of the *PvMSP3α* by *Hha*I showed the same restriction patterns for the first three attacks, whereas the fifth attack was clearly different, suggesting that the first three attacks were likely due to the same parasite strain, whereas the last attack was from a different parasite strain (Fig. 1b).

Since the effectiveness of PQ for radical cure of vivax malaria is influenced by host CYP2D6 activity, we wanted to determine whether the failure of PQ in this case might be linked to CYP2D6 genotypes suggestive of poor metabolizer of PQ. The single nucleotide polymorphisms (SNPs) in *CYP2D6* were determined by PCR amplification of the full-length *CYP2D*6 coding region using a high-fidelity enzyme and sequencing of the PCR products, similar to a method described earlier [28]. Primary PCR was performed using primers P1 (5′-CTGGCAGCACAGTCAACA-3′) and P2 (5′-TTTGTCTTCCGTTTTGGG-3′), while nested reactions were done with primers N1 (5′-ATAAGGGAAGGGTCACGC-3′) and N2 (5′-GGCAAGGGTAACTGACATCT-3′). The following PCR conditions were used: initial denaturing at 95 °C for 3 min, 35 cycles of 95 °C for 15 s, 53 °C (58 °C for nested reactions) for 15 s, and 72 °C for 5 min, and final extension at 72 °C for 5 min. PCR and sequencing of *CYP2D6* detected mutations 214G > C, 221C > A, 223C > G, 227 T > C, 232G > C, 233A > C, 245A > G, 310G > T, 745C > G, 842 T > G, 1662G > C, 2851C > T, 3385A > C, 3585G > A, 3791C > T, 4181G > C, and 4482G > A, which are classified as *2A, and mutations 100C > T, 310G > T, 842 T > G, 1038C > T, 1662G > C, 2098A > G, 3385A > C, 3583A > G, 4125G > C, 4129C > G, 4132A > G, 4134 T > C, 4156C > T, 4157A > C, 4159G > C, 4165 T > G, 4167 T > C, 4168G > A, 4169C > G, 4170 T > C, 4173C > T, and 4181G > C, which are classified as *36. According to the CYP2D6 allele naming database (www.Pharmvar.org/gene/CYP2D6), the patient’s CYP2D6 genotype corresponds to a *2A/*36 allele variant. CYP2D6*2A is predicted to be functionally normal (score 1), but *36 is non-functional (score 0). Thus, the overall genotype activity score was 1, suggesting that this patient’s CYP2D6 was an impaired PQ metabolizer phenotype [21]. Real-time PCR was performed to determine the *CYP2D6* gene copy number using a previously described method [29], and the result showed that *CYP2D6* gene in this patient was a single copy.

## Discussion and conclusions

We documented a *P. vivax* infection acquired in West Africa producing four relapses with the last relapse occurring at 491 days after the first attack. This study suggests that although vivax malaria is considered to be exceedingly rare in West Africa because of predominance of Duffy-negativity in the local human population, there is a considerable hazard for Duffy-positive travelers to acquire relapsing malaria parasites in this region. This malaria case provides evidence indicating that *P. vivax* strains from West Africa may produce multiple relapses extending beyond one year but less than two. The relapse patterns of *P. vivax* strains from different geographical regions can vary significantly, from the tropical ‘frequent-relapse phenotype’ to the temperate “long-latency” phenotype’ [30]. The relapse intervals of this Western African strain ranged from 58 days to 232 days. This is quite different from the relapsing patterns of *P. vivax* strains from the tropical Pacific region such as the Chesson strain, which relapse quickly but typically do not extend beyond one year [30]. This patient stayed the entire time after returning from Ghana in Guangxi, a malaria-free area after 2012 [31], thus the possibility of new infections was highly improbable but could not be ruled out since Guangxi borders with Vietnam and Yunnan, where very low-level transmission of *P. vivax* occurred. Furthermore, genotyping of the parasites by PCR-RFLP of *PvMsp3a* gene indicates that the first two relapses had the same genotype as the primary infection, although the parasite from the fourth relapse had a different genotype. This suggests that the initial inoculum might include more than one strain of *P. vivax* as sporozoites from one oocyst in the mosquito may comprise sibling progeny of a single gametocyte mating event, or that he had multiple infections during his stay in Ghana. It is not uncommon that relapses and primary infections are genotypically different and relapses often result from activation of heterologous hypnozoites [32, 33].

Despite the fact that this Chinese patient received PQ treatment each time he had acute *P. vivax* infection, PQ therapy failed to prevent subsequent relapses. The excessive blood schizonticides given to this patient were not rational and did not prevent relapses. There are a number of possible reasons for the PQ treatment failures. One possibility is that the 8-day PQ treatment is able to prevent relapses for the temperate *P. vivax* strain [34] and the subtropical strains [35] in China, but may be ineffective against West African *P. vivax*. It is noteworthy that although the daily adult dose for the 8-day PQ regimen (22.5 mg) is higher than for the standard 14-day regimen (15 mg/day), the total PQ dosage for the 8-day course (180 mg) is lower than for the 14-day course (210 mg). In addition, since the 8-day PQ regimen (0.375 mg/kg/day) is calculated for adults weighing 60 kg, this patient was also slightly under-dosed (0.32 mg/kg/day). Currently, there are no clinical studies testing the effectiveness of PQ treatment against relapses of West African *P. vivax* strains. In Shanglin County Hospital, of 105 *P. vivax* patients who received the 8-day PQ regimen between 2013 and May 2019, four patients (3.8%) had relapses, suggesting that the 8-day PQ regimen worked at least partially for preventing relapses of *P. vivax* parasites. Another possibility for the PQ failure lies in the patient’s CYP2D6 enzyme, which is known to metabolize PQ to its active metabolite(s) for the hypnozoitocidal activity of PQ [16]. This patient has a CYP2D6 *2A/*36 genotype, which corresponds to being an impaired PQ metabolizer phenotype. CYP2D6 intermediate and poor metabolizer phenotypes have been linked to poor treatment outcome of PQ anti-relapse therapy in clinical trials [15, 25]. Subjects with an activity score phenotype of 1.0 for CYP2D6 have shown variable responsiveness to PQ therapy against relapse of *P. vivax*; among the 7 patients evaluated by Bennett et al., none relapsed, whereas among the 22 patients evaluated by Baird et al., 12 relapsed [15, 25]. Although our patient may seem to have been administered a normally inadequate regimen of primaquine, he received it four times delivering a total dose of 720 mg (10.3 mg/kg) that failed to prevent relapse. Interestingly, a case report showed that repeated relapses of vivax malaria acquired in Papua New Guinea also were likely attributed to the patient’s intermediate metabolizer phenotype of the CYP2D6 allele (*5/*41) [36]. The current case of *P. vivax* malaria acquired in West Africa adds further support to the importance of the CYP2D6 polymorphisms for the effectiveness of the PQ anti-relapse therapy. With the demonstrated efficacy of a single-dose tafenoquine in preventing relapses of vivax malaria [37] and the seemingly lack of effects of CYP2D6 on tafenoquine [38], tafenoquine may be the choice of anti-relapse medicine in G6PD normal patients. It nonetheless remains uncertain whether tafenoquine is CYP2D6-dependent or not.

## Data Availability

All relevant data and materials are included in the manuscript.

## Abbreviations

ACT: Artemisinin-based combination therapy
CDC: Center for Disease Control
CQ: Chloroquine
CYP: Cytochrome P450
DOT: Directly observed therapy
G6PD: Glucose-6-phosphate dehydrogenase
IV: Intravenous
PQ: Primaquine
RFLP: Restriction fragment length polymorphism
